# A Nutritional Metabolism Related Prognostic Scoring System for Patients With Newly Diagnosed Osteosarcoma

**DOI:** 10.3389/fnut.2022.883308

**Published:** 2022-04-28

**Authors:** Longqing Li, Zhuangzhuang Li, Xuanhong He, Yang Wang, Minxun Lu, Taojun Gong, Qing Chang, Jingqi Lin, Yi Luo, Li Min, Yong Zhou, Chongqi Tu

**Affiliations:** ^1^Department of Orthopedics, Orthopedic Research Institute, West China Hospital, Sichuan University, Chengdu, China; ^2^Bone and Joint 3D-Printing and Biomechanical Laboratory, Department of Orthopedics, West China Hospital, Sichuan University, Chengdu, China

**Keywords:** osteosarcoma, nutrition, metabolism, prognosis, hematology

## Abstract

Osteosarcoma is a primary malignant bone tumor with high metastatic potential. To date, achieving long-term survival of osteosarcoma patients remains a difficult task. Metabolic reprogramming has emerged as a new hallmark of cancer. However, studies on the prognostic value of hematological markers related to nutritional and metabolism in cancer patients are limited and contradictory. In this retrospective study, we extensively collected 16 hematological markers related to nutritional and metabolism in 223 osteosarcoma patients. A nutritional metabolism related prognostic scoring system (NMRS) in patients with osteosarcoma was constructed by least absolute contraction and selection operator (LASSO) cox regression analysis. Compared with individual hematological indicators, NMRS has stronger predictive power (training set: 0.811 vs. 0.362–2.638; validation set: 0.767 vs. 0.333–0.595). It is an independent prognostic factor for the survival of patients with osteosarcoma [HR: 1.957 (1.375–2.786) training set; HR: 3.146 (1.574–6.266) validation set]. NMRS-based nomograms have good and stable predictive power. NMRS facilitates further risk stratification of patients with the same clinical characteristics.

## Introduction

Osteosarcoma is the most common primary malignant bone tumor, accounting for 20–40% of all bone tumors (1, 2). Tumors tend to occur in children and adolescents and have a high metastatic potential. The 5-year survival rate of patients with standard treatment is about 60–70% (3). However, approximately 15–20% of patients have developed metastases at initial diagnosis, and the five-year survival rate is significantly reduced, so far there is no effective treatment regimen (4–6). In addition, insensitivity to chemotherapy and tumor recurrence are also important factors leading to significantly reduced patient survival (7, 8). These clinical features can identify high-risk patients and aid in treatment planning (9). However, the progression of the disease may be distinct in patients with similar clinical features. Therefore, more factors need to be considered to facilitate precision treatment.

Recently, researchers have made many efforts in developing biomarkers that can more accurately predict the prognosis of patients and developed many biomarkers with potential, such as non-coding RNA (NcRNA), circulating tumor cell (CTC), and circulating tumor DNA (ctDNA) (10–12). However, these markers have not been applied to clinical work due to reasons such as the cost of testing, strict technical requirements for biopsy, etc. Fortunately, studies have shown that preoperative hematological markers such as neutrophil to lymphocyte ratio (NLR), platelet to lymphocyte ratio (PLR) or hematology-based scores such as Glasgow Prognostic Score (GPS), Controlling Nutritional Status (COUNT) show great potential in predicting the prognosis of cancer patients (13–15). Compared with NcRNA, CTC, and ctDNA, most of these hematological markers originate from routine examinations of patients on admission without the need for additional testing costs. Many recent studies have confirmed the value of these markers in predicting survival in cancer patients, including osteosarcoma (16–18).

Studies over the past decade have shown that cancer cells can promote their survival by reprogramming metabolic pathways, and therefore, metabolic reprogramming is also considered to be one of the hallmarks of cancer (19, 20). Local tumors can even impair antitumor immunity by affecting host metabolism through cachexia (21). However, studies on the ability of hematologic metabolic markers to predict the prognosis of cancer patients are contradictory and limited compared with hematologic inflammatory markers (22).

In this study, we collected the hematological markers related to metabolism and explored the significance of disturbances in these markers in patients with osteosarcoma. Through iterative least absolute contraction and selection operator (LASSO) COX proportional hazards regression analysis we constructed nutritional metabolism related prognostic scoring system (NMRS) and assessed the predictive power of the scores through multiple dimensions. In addition, we also explored the superiority and limitations of NMRS by comparing it with existing scoring systems and clinical features.

## Patients and Methods

### Patients

We reviewed the clinical data of osteosarcoma patients who visited the Musculoskeletal Tumor Center of West China Hospital from January 2016 to January 2021. Patients were screened with the following inclusion and exclusion criteria. inclusion criteria: (1) patients with histopathologically confirmed high-grade osteosarcoma; (2) patients with blood routine, liver and kidney function, coagulation function tests before neoadjuvant chemotherapy; (3) patients who completed standard osteosarcoma treatment regimen at West China Hospital. Exclusion criteria: (1) patients with concomitant metabolic disease; (2) patients with concomitant hematological diseases; (3) patients with other malignancies; (4) patients who were previously misdiagnosed or mistreated. Finally, 223 patients who met the inclusion criteria and passed the exclusion criteria were included in the study. A standard follow-up principle was developed to follow each patient, with the last follow-up date being January 2022. The follow-up principle: reexamination every 3 months within 1 year after surgery; reexamination every 4 months 1–2 years after surgery; reexamination every 5 months 2–3 years after surgery; reexamination every 6 months 3–5 years after surgery; reexamination every year more than 5 years after surgery. All patients were randomly assigned to the training set (*n* = 156, 70%) vs. the validation set (*n* = 67, 30%).

### Data Collection and Processing

The following data were extracted from each patient’s first blood routine, liver and kidney function: Red blood cells (RBC), Red blood cell specific volume (HCT), Hemoglobin (HB), Lymphocyte count (LYMPH#), albumin (A), Globulin (G), Glucose (GLU), Cholesterol (TCH), Triglycerides (TG), High density liptein cholesterol (HDL-C), Low Density Lipoprotein (LDL), Total bilirubin (TBIL), Indirect bilirubin (IBIL), Direct bilirubin (DBIL). The calculation formulas of AGR and PNI are as follows. AGR = A/G; PNI = A + 0.005 × LYMPH#. CONUT was calculated following previous studies. Supplementary Table 1 provides the specific calculation formula. In the overall cohort, the receiver operating curve was used to find the optimal cutoff for continuous variables and continuous variables were transformed into binary variables based on the cutoff.

### Construction of Nutritional Metabolism Related Prognostic Scoring System

First, univariate Cox regression analysis was used to screen for prognostic related indicators. The LASSO regression analysis was performed 1,000 times on the training set using the screened hematological markers to build the model. Hematological markers that were retained at high frequencies in the 1,000 times LASSO regression analyses were sequentially included in the cox model. The model when AUROC peaked was considered the best model. NMRS risk scores were calculated for each patient including the validation set based on markers and coefficients in the model.

### Evaluation of the Value of Nutritional Metabolism Related Prognostic Scoring System

First, we contrasted the differences in predictive ability between NMRS scores and its constituent markers using ROC analysis. Subsequently, with the “survivalROC” package, we determined the optimal cutoff value for the NMRS score. All patients were divided into two groups according to the cutoff value and differences in overall survival between the two groups of patients were assessed using Kaplan–Meier (KM) survival analysis. Given that the effects of continuous variables on risk can be non-linear, ignoring such non-linear effects can interfere with the results, risk scores were analyzed using restricted cubic splines. To further clarify the value of NMRS, we also plotted time-dependent ROC curves to explore changes in NMRS predictive ability over time and contrasted with clinical characteristics. Subsequently, through multivariate cox regression analysis, we explored whether NMRS was an independent prognostic factor for overall survival in patients with osteosarcoma. Finally, to explore the stability of the predictive power of NMRS scores, we set up subgroups based on clinical characteristics and explored the predictive power of NMRS in each subgroup.

### Construction and Evaluation of the Nomogram

To facilitate the clinical application of NMRS, we combined NMRS with clinical features to construct a NMRS-based nomogram. The discrimination ability and accuracy of nomograms were assessed by C-index and calibration curve, respectively. To clarify whether the predictive power of nomogram prediction is stable, we predicted the overall survival of patients in the validation set using nomograms and assessed it using the C-index and the calibration curve. Finally, whether the application of nomograms can bring about clinical net benefit and net reduction was evaluated by decision curve analysis.

### Relationship Between Nutritional Metabolism Related Prognostic Scoring System and Clinical Characteristics

We analyzed differences in NMRS scores across clinical characteristics to assess the relationship between NMRS and clinical characteristics. In addition, we simply combined NMRS with important clinical characteristics [tumor metastasis status, pathological fracture status, Body Mass Index (BMI)] to divide patients into multiple groups, and plotted KM survival curves to assess the difference in survival between different groups of patients to explore whether NMRS can further distinguish patients with the same clinical characteristics.

### Statistical Analysis

Descriptive statistics were used to assess any differences between datasets using the *t*-test or Mann–Whitney *U* test for continuous variables and the chi-square test or Kruskal–Wallis test for categorical variables. All statistical analyses were conducted using R software, version 4.1.0 (Institute for Statistics and Mathematics, Vienna, Austria). *P* values < 0.05 were considered to indicate statistical significance.

## Results

### Patient Characteristics

The study included 131 male and 92 female with a total of 223 patients. Median follow-up of patients was 3.4 ± 0.4 years. As shown in Table 1, variables did not differ significantly between the training set and the validation set. The mean age of the patients in the training and validation sets were 21.3 and 22.5, respectively. Metastasis occurred in 17.9% (23) of patients in the training set and 16.4% (11) in the validation set. Only a very small proportion of patients had tumors located in non-extremity sites (7 patients in training set; 2 patients in validation set). Seventeen patients in the training set and 8 patients in the validation set were presenting for pathological fractures. In addition, Table 1 shows the markers and their coefficients that make up the NMRS. Supplementary Table 2 provides the optimal cutoff values for each marker.

**TABLE 1:** Differences in the distribution of all variables between the training set and the validation set and the respective coefficients of the 9 hematological markers that make up the NMRS.

	Train (*N* = 156)	Test (*N* = 67)	*P*-value	Coefficient
**OS time**				Not applicable
Mean (SD)	1,030 (545)	975 (576)	0.524	
**OS**				Not applicable
Alive	105 (67.3%)	42 (62.7%)	0.608	
Died	51 (32.7%)	25 (37.3%)		
**Gender**				Not applicable
Male	93 (59.6%)	38 (56.7%)	0.799	
Female	63 (40.4%)	29 (43.3%)		
**Age**				Not applicable
Mean (SD)	21.3 (12.3)	22.5 (12.4)	0.483	
**Metastasis status**				Not applicable
No	128 (82.1%)	56 (83.6%)	0.933	
Yes	28 (17.9%)	11 (16.4%)		
**Tumor site**				Not applicable
Extremities	149 (95.5%)	65 (97.0%)	0.88	
Non-extremities	7 (4.5%)	2 (3.0%)		
**Pathological fracture**				Not applicable
No	139 (89.1%)	59 (88.1%)	1	
Yes	17 (10.9%)	8 (11.9%)		
**PNI**				Excluded
High	98 (62.8%)	44 (65.7%)	0.799	
Low	58 (37.2%)	23 (34.3%)		
**AGR**				−0.497
High	71 (45.5%)	33 (49.3%)	0.714	
Low	85 (54.5%)	34 (50.7%)		
**CONUT**				0.354
High	57 (36.5%)	25 (37.3%)	1	
Low	99 (63.5%)	42 (62.7%)		
**RBC**				Excluded
High	125 (80.1%)	53 (79.1%)	1	
Low	31 (19.9%)	14 (20.9%)		
**HB**				Excluded
High	101 (64.7%)	47 (70.1%)	0.53	
Low	55 (35.3%)	20 (29.9%)		
**HCT**				Excluded
High	106 (67.9%)	48 (71.6%)	0.697	
Low	50 (32.1%)	19 (28.4%)		
**Total bilirubin**				Excluded
High	15 (9.6%)	13 (19.4%)	0.0716	
Low	141 (90.4%)	54 (80.6%)		
**Direct bilirubin**				Excluded
High	94 (60.3%)	48 (71.6%)	0.142	
Low	62 (39.7%)	19 (28.4%)		
**Indirect bilirubin**				Excluded
High	73 (46.8%)	41 (61.2%)	0.0679	
Low	83 (53.2%)	26 (38.8%)		
**Albumin**				−0.286
High	117 (75.0%)	51 (76.1%)	0.993	
Low	39 (25.0%)	16 (23.9%)		
**Globulin**				0.417
High	92 (59.0%)	38 (56.7%)	0.869	
Low	64 (41.0%)	29 (43.3%)		
**Glucose**				0.562
High	39 (25.0%)	24 (35.8%)	0.138	
Low	117 (75.0%)	43 (64.2%)		
**Triglycerides**				0.596
High	43 (27.6%)	20 (29.9%)	0.853	
Low	113 (72.4%)	47 (70.1%)		
**Cholesterol**				−1.127
High	99 (63.5%)	42 (62.7%)	1	
Low	57 (36.5%)	25 (37.3%)		
**HDL**				−0.188
High	96 (61.5%)	40 (59.7%)	0.914	
Low	60 (38.5%)	27 (40.3%)		
**LDL**				0.901
High	82 (52.6%)	36 (53.7%)	0.989	
Low	74 (47.4%)	31 (46.3%)		

### Construction of Nutritional Metabolism Related Prognostic Scoring System

**FIGURE 1:**
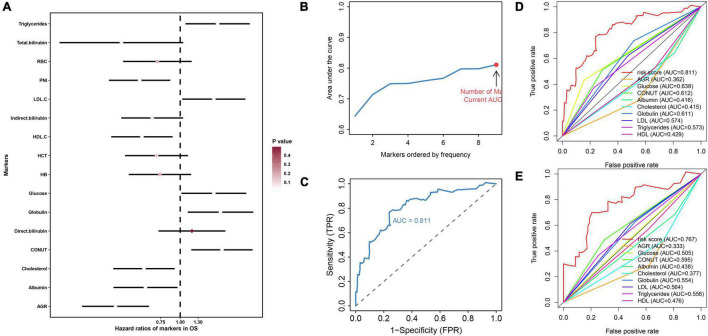
The construction process of NMRS and the comparison between NMRS and individual hematological markers in terms of predictive ability. **(A)** Forest plot presented the results of univariate cox regression analysis of 16 hematological markers in the overall cohort. Where the smaller the *p* value, the closer the color of the diamond of the marker is to white; **(B,C)** With the continuous inclusion of the marker with high frequency retained in LASSO regression analysis into the model, the AUC value of the model continues to rise. When the 9th marker is included in the model, the AUC value of the model reaches the highest value, and this model is NMRS; **(D)** ROC curves of the predictive ability of NMRS and individual hematological markers in the training set; **(E)** ROC curves of the predictive ability of NMRS and individual hematological markers in the validation set.

As described in the “Patients and Methods” section, based on the overall cohort, by univariate cox regression analysis, we identified 10 markers with prognostic value and used them for further analysis (Figure 1A). Subsequently, LASSO regression analysis was performed 1,000 times in the training cohort using prognostic markers to determine 9 hematological markers with high frequency retained and constructed NMRS (Figures 1B,C). The coefficients for each marker in the NMRS are shown in Table 1, and the NMRS was calculated for each patient based on these coefficients.

### Evaluation of Prognostic Value of Nutritional Metabolism Related Prognostic Scoring System

**FIGURE 2:**
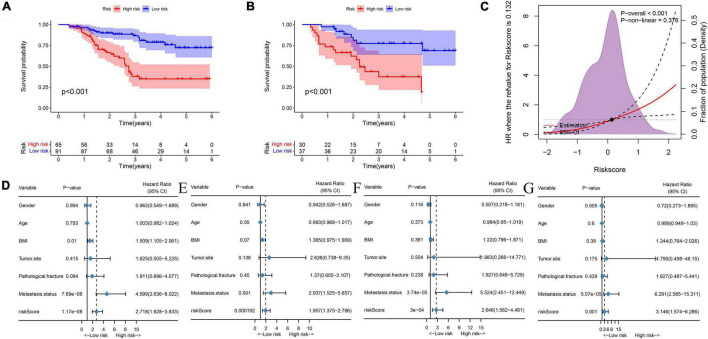
NMRS has independent prognostic value in both the training and validation sets. **(A)** KM survival curves showing overall patient survival in the training set; **(B)** KM survival curves showing overall patient survival in the training set; **(C)** The overall cohort patient NMRS score and overall survival risk, restricted cubic splines indicated that the effect of NMRS as a continuous variable on overall survival risk was linear; **(D)** Results of univariate cox regression analysis of NMRS and clinical characteristics in the training set; **(E)** Results of multivariate cox regression analysis of NMRS and clinical characteristics in the training set; **(F)** Results of univariate cox regression analysis of NMRS and clinical characteristics in the validation set; **(G)** Results of multivariate cox regression analysis of NMRS and clinical characteristics in the validation set.

First, by ROC analysis, we contrasted NMRS with every hematological marker to explore whether there was an advantage in NMRS. As shown by Figures 1C,D, NMRS has the largest area under the curve (AUC) in both training and validation sets and is significantly higher than other markers (training set: 0.811 vs 0.362–2.638; validation set: 0.767 vs 0.333–0.595). The optimal cutoff value of NMRS was 0.132, and patients were divided into two groups based on the cutoff value, and the overall survival of low-risk patients was significantly longer than that of high-risk patients in both the training and validation sets (Figures 2A,B, *p* < 0.001). Finally, results from restricted cubic splines show that the effect of NMRS on prognosis is linear (Figure 2C, *p* = 0.376).

**FIGURE 3:**
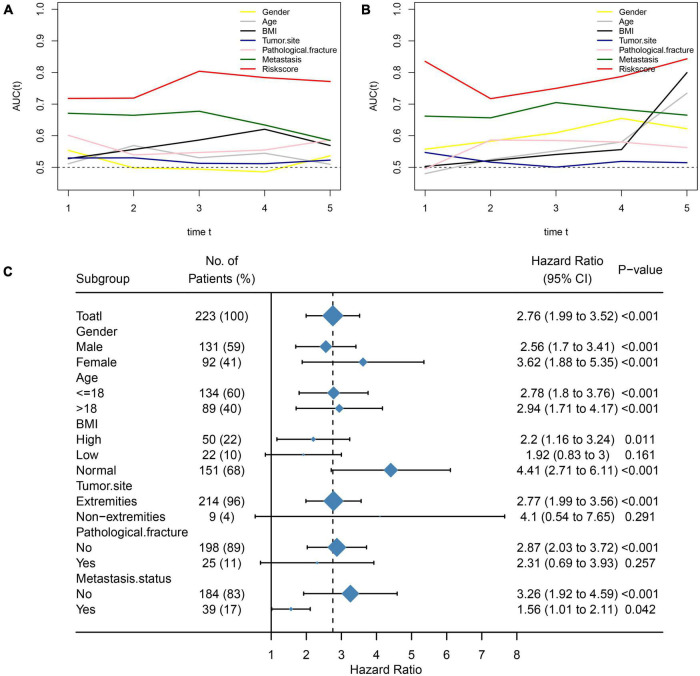
Comparison of NMRS with clinical features and stability of NMRS prediction ability. **(A,B)** Time-dependent ROC curves for NMRS vs. clinical characteristics in terms of predictive ability (training set and validation set); **(C)** Forest plots showing the predictive power of NMRS in various subgroups.

We further assessed whether the prognostic value of NMRS would be influenced by clinical characteristics. As shown in Figures 2D–G, multivariate cox regression analysis revealed that only NMRS score and metastatic status were independent prognostic factors in both training and validation sets [NMRS: HR: 1.957 (1.375–2.786) training set; HR: 3.146 (1.574–6.266) validation set]. As shown in Figures 3A,B, the time-dependent ROC curve showed that the predictive ability of NMRS did not decrease with time.

We also assessed the stability of the predictive ability of NMRS by the subgroup analysis. It can be seen that the predictive ability of NMRS has good stability, has prognostic value in most of subgroup, and is limited only in three group of patients with small sample size (Figure 3C).

### Construction and Validation of Nutritional Metabolism Related Prognostic Scoring System-Based Nomograms

**FIGURE 4:**
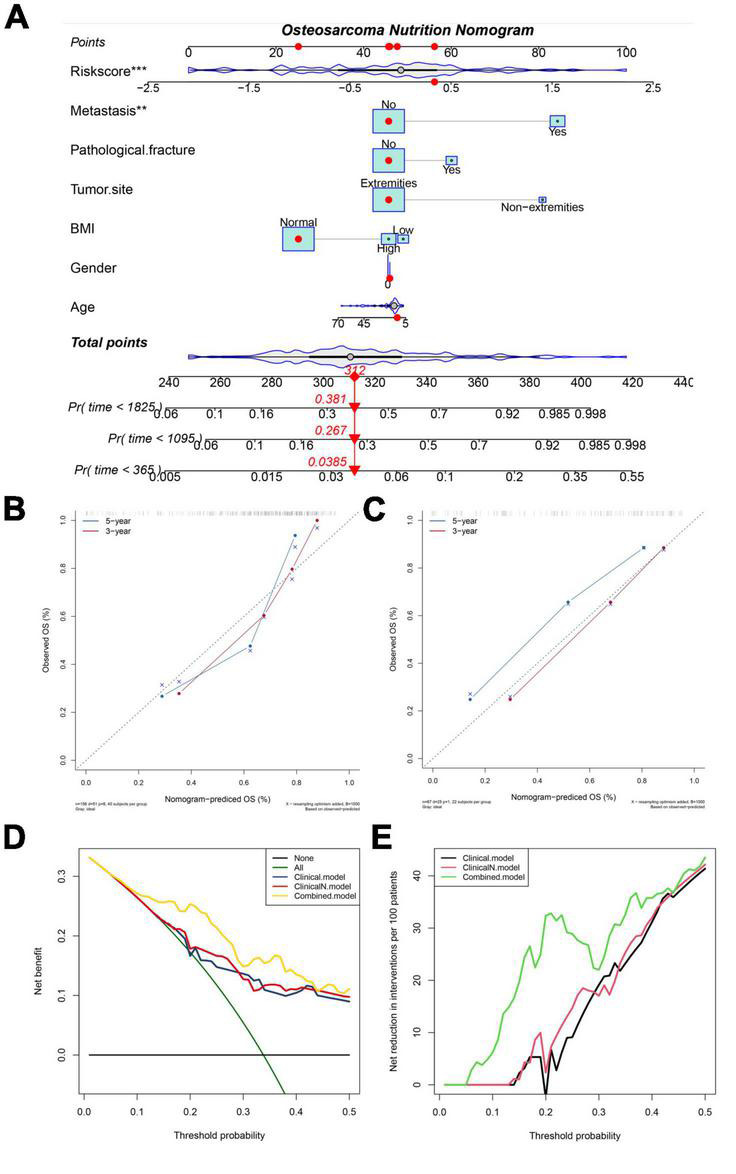
NMRS-based nomograms have good predictive ability and predictive stability. **(A)** NMRS-based nomogram, in which NMRS with tumor metastasis status is the two most important factors; **(B,C)** Calibration curve of NMRS nomogram for predicting 3-year vs. 5-year overall survival in patients with osteosarcoma (training set and validation set); **(D)** Decision curve analysis of the NMRS nomogram, which only complements BMI to clinical features yielded little clinical net benefit (clincalN). When NMRS is introduced into clinical features it yields definite clinical net benefit (Combined); **(E)** The introduction of BMI into clinical features produced little clinical net reduction, whereas the introduction of NMRS brought clinical net reduction with certainty.

To improve the accuracy of the prediction of overall survival in osteosarcoma, we constructed a nomogram combining NMRS with clinical features in the training set. As shown in Figure 4A, NMRS and tumor metastasis status were the two most important components in the nomogram. NMRS has the largest scoring interval, ranging from 0 to 100. The C-index of the nomogram was 0.80, suggesting that the nomogram has a good discriminative ability. The results of the calibration curve show that the nomogram also has good accuracy (Figure 4B). The nomogram also has a good performance in the validation set with a C-index of 0.80, suggesting that the predictive ability of the nomogram is stable (Figure 4C). The results of decision curve analysis showed that the introduction of NMRS on the basis of clinical characteristics can bring about clinical net benefit vs. net reduction (Figures 4D,E).

### Association Between Nutritional Metabolism Related Prognostic Scoring System and Clinical Features

**FIGURE 5:**
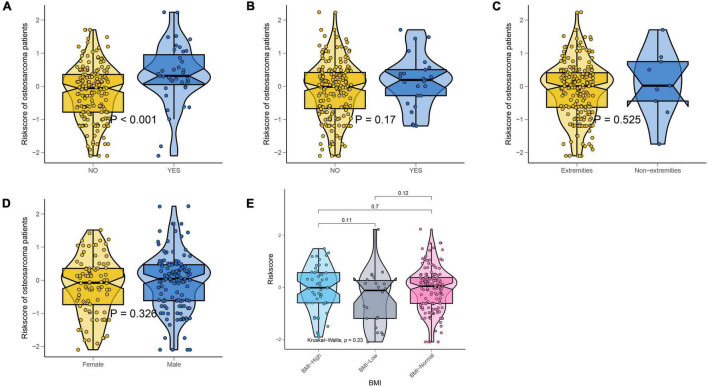
NMRS was only associated with metastatic status. **(A)** Violin plots showing differences in the distribution of NMRS among patients with different metastatic status; **(B)** Violin plots showing differences in the distribution of NMRS among patients with different pathological fracture state; **(C)** Violin plots showing differences in the distribution of NMRS among patients with different tumor location; **(D)** Violin plots showing differences in the distribution of NMRS among patients with different gender; **(E)** Violin plots showing differences in the distribution of NMRS among patients with different BMI.

**FIGURE 6:**
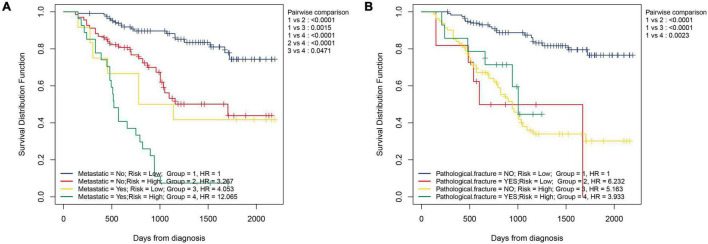
NMRS can further identify patients with different risks on the basis of clinical characteristics. **(A)** Two-factor KM survival curves considering the risk of NMRS and the status of tumor metastasis; **(B)** Two-factor KM survival curves considering the risk of NMRS and the status of pathological fracture.

We also explored whether there were differences in NMRS scores between different clinical subgroups. As shown in Figure 5, the NMRS scores were lower in the non-metastaticgroup, and there was no significant difference in the NMRS scores among the remaining subgroups. Finally, the results of two-factor survival analysis showed that NMRS can be further risk-stratified from patients with the same clinical characteristics (Figure 6). Based on NMRS and tumor metastasis status, patients were divided into four groups, and the overall survival of patients with high NMRS risk was significantly higher than that of patients with low NMRS risk in the non-metastatic group (*p* < 0.0001), even close to that of patients with low NMRS risk in tumor metastasis (*p* > 0.05). Even among patients in the tumor metastasis group, there were differences in the overall survival of patients with different NMRS risks (*p* = 0.0471). When patients were divided into four groups according to pathological fracture status and NMRS risk, overall survival was significantly lower in patients with high NMRS risk among patients with non-pathological fractures (*p* < 0.0001).

### Prognostic Value of Individual Hematological Markers

**FIGURE 7:**
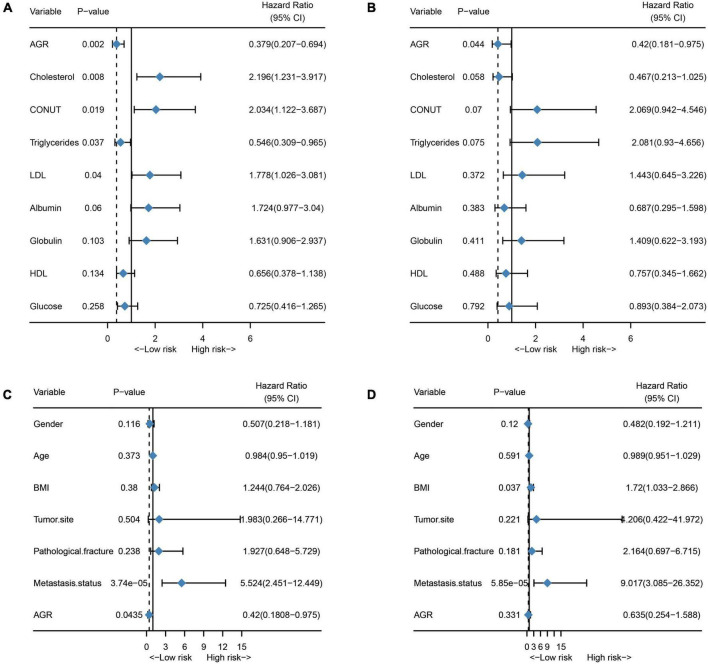
Prognostic value of individual hematological markers and AGR in the training and validation sets. **(A)** Univariate cox regression analysis results of a single hematological marker in the training set; **(B)** Univariate cox regression analysis results of a single hematological marker in the validation set; **(C)** Univariate cox regression analysis of AGR in the validation set; **(D)** Multivariate cox regression analysis of AGR in the validation set.

Finally, we assessed the prognostic value of the 9 hematological markers that constitute NMRS in osteosarcoma. As shown in Figure 7A, the results of univariate cox regression analysis showed that AGR, TCH, COUNT, TG and LDL had significant prognostic value in the training set. However, only the prognostic value of AGR remained statistically significant in the validation set (Figure 7B). We further explored whether AGR is an independent prognostic factor in osteosarcoma patients in the validation set. Unfortunately, the results of multivariate cox regression analysis indicated that AGR was not an independent prognostic factor in osteosarcoma patients (Figures 7C,D).

## Discussion

Although limb salvage surgery for osteosarcoma has been continuously improved in recent years, and most patients can obtain satisfactory limb function after surgery, it is still a difficult task for osteosarcoma patients to achieve survival (1, 24, 25). Early identification of high-risk patients and development of personalized treatment options are expected to improve the prognosis of patients (26). Unfortunately, in osteosarcoma, no new prognostic marker has been truly applied to clinical work except for clinical features such as metastatic status and tumor necrosis rate (9). Although recent studies have shown the potential of NcRNA, CTC with ctDNA as a tool for early detection, postoperative monitoring of cancer patients (23, 27–29). However, because the concentration of CTC and ctDNA collected from patients is usually very low. In addition, standard methods for isolation, enrichment, or detection of NcRNA, CTC, and ctDNA are lacking (30, 31). Therefore, there is still a certain distance between NcRNA, CTC and ctDNA for real clinical application. In contrast, because most of the hematological markers are derived from routine examinations such as blood routine and liver and kidney function, no additional testing costs are required. Moreover, the value of hematological markers in predicting the prognosis of cancer patients has been generally accepted, for example, NLR has been written into the 2020 European Association of Urology Guidelines on Upper Urinary Tract Urothelial Carcinoma (32). However, the prognostic value of indicators such as TG, HDL and LDL in cancer patients has not been determined. Therefore, we extensively collected some hematological markers that may reflect nutritional status and metabolic reprogramming in cancer patients and constructed NMRS. As expected, NMRS demonstrated greater stability and predictive power. With the exception of NMRS, none of the hematological markers had independent prognostic ability in both the training and validation sets. In addition, NMRS has better discrimination ability than clinical features such as tumor metastasis status, especially in identifying low-risk patients.

The lipid metabolism plays an important role in cancer metabolic reprogramming. Cancer cells, as well as other cell types in the tumor microenvironment, utilize various methods to obtain lipids and extensively rewrite their metabolism (33). Studies have shown that cancer stem cells maintain their stem cell characteristics through lipid metabolism (34). In addition, alterations in lipid metabolism can impair antitumor immunity and promote iron death escape (35, 36). Several large-scale lipidomics studies have provided compelling evidence for the potential of lipids as prognostic biomarkers for cancer (33, 37, 38). In this study, lipid metabolic markers were the most important cornerstone of NMRS, with TCH, TG, HDL, and LDL coefficients of −1.127, 0.596, −0.188, and 0.901, respectively, which indicated that higher TCH and HDL was associated with better prognosis, while triglyceride and LDL were the opposite.

Serum albumin and globulin reflect the nutritional and inflammatory status of individuals and have shown potential prognostic value in a variety of tumors (39–41). On the one hand, albumin reflects the nutritional status of individuals, and in general, patients with poor nutritional status have a higher risk of postoperative complications, which may greatly shorten the survival time of cancer patients (42). On the other hand, decreased serum albumin may be due to increased capillary permeability caused by cancer-related inflammation resulting in albumin escape into the interstitium and absorption by cancer cells, decomposition and utilization (43, 44). In addition, globulin is considered a pro-inflammatory protein and has been shown to be associated with poor prognosis in cancer patients (45). In this study, the coefficients of albumin, globulin and AGR were −0.286, 0.417 and −0.497, respectively, confirming the previous conclusions (46–48).

Although the mechanisms by which high glucose promotes cancer aggressiveness vary by cancer type, it is generally accepted that high glucose is associated with poor prognosis in cancer patients. Studies have shown that abnormal elevated glucose that does not reach the diagnostic level of diabetes is also associated with poor prognosis in cancer patients (49). A high glucose environment leads to the up-regulation of aerobic glycolysis (Warburg effect) -related pathways in cancer cells. In addition, glucose can also activate a variety of signaling pathways involved in cell proliferation, metastatic capacity, and chemoresistance, including ERK, STAT3, and NF-κB (50, 51). The coefficient for glucose in this study was 0.562, indicating that elevated glucose is associated with poor prognosis in osteosarcoma patients. Notably, to our knowledge, the prognostic value of glucose in osteosarcoma patients has not been previously investigated. Finally, CONUT consists of lymphocytes and albumin together with cholesterol, so it is not difficult to understand that CONUT is associated with the prognosis of cancer patients. The coefficient of CONUT in NMRS was 0.354, which is consistent with previous findings (52).

As shown by Figure 1, the results of our ROC analysis showed that there was no significant difference in the prognostic value of individual hematological markers between the training and validation sets. The AUC values of NMRS were higher than all the individual hematological markers. In addition, NMRS combines all of the above indicators and assigns coefficients to each indicator, which comprehensively reflects these indicators and better and more stably represents the nutritional and metabolic status of patients. In fact, we also performed further analysis of individual hematological marker. Our results showed that only AGR had some prognostic value in both the training set and the validation set. In multivariate cox regression analysis combined with clinical characteristics, none of the individual hematological parameters were independent prognostic factors for overall survival of osteosarcoma patients in both the training and validation sets. These results further demonstrate the superiority of NMRS. We believe that NMRS with higher and more stable predictive ability is more likely to be applied and promoted in clinical practice.

However, it must be acknowledged that our study has certain limitations. Despite the training and validation sets of our study setup, all patients were from one clinical institution and the study was retrospective and not blinded, so there may have been a selection bias. At the same time, there were only two osteosarcoma patients over 60 years of age in the included population, so the use of NMRS in the elderly requires caution. Moreover, some patients may have problems with insufficient follow-up time. In addition, although the predictive ability of NMRS is higher than that of individual hematological markers, the computational method of NMRS is more complex. Finally, the metabolic nutrition-related indicators included in the study were derived from blood routine and liver function only. Some important indicators that reflect the patient’s nutritional status, such as nutrients or dietary intake, are neglected. Therefore, well-designed prospective randomized controlled studies are needed to validate our conclusions. Finally, we believe that further studies are needed to explore the relationship between NMRS and dietary intake in patients.

## Conclusion

Our study show the prognostic value of NMRS in osteosarcoma. Compared with individual hematological markers, NMRS has predictive ability and predictive stability. NMRS-based nomogram also have good predictive accuracy.

## Data Availability Statement

The raw data supporting the conclusions of this article will be made available by the authors, without undue reservation.

## Ethics Statement

The studies involving human participants were reviewed and approved by Ethics Committee of West China Hospital of Sichuan University. Written informed consent to participate in this study was provided by the participants’ legal guardian/next of kin.

## Author Contributions

LL, YZ, and CT designed the study. LL, ZL, XH, YW, JL, and QC jointly collected and managed the data. LL and TG drafted the manuscript. ML, YL, and LM reviewed and corrected the manuscript. YZ and CT oversaw the entire research process. All authors contributed to the article and approved the submitted version.

## Conflict of Interest

The authors declare that the research was conducted in the absence of any commercial or financial relationships that could be construed as a potential conflict of interest.

## Publisher’s Note

All claims expressed in this article are solely those of the authors and do not necessarily represent those of their affiliated organizations, or those of the publisher, the editors and the reviewers. Any product that may be evaluated in this article, or claim that may be made by its manufacturer, is not guaranteed or endorsed by the publisher.
