# Supplementary material for: A Nutritional Metabolism Related Prognostic Scoring System for Patients With Newly Diagnosed Osteosarcoma
Source: Front Nutr. 2022 Apr 28;9:883308. doi: 10.3389/fnut.2022.883308 (PMC9096723; doi:10.3389/fnut.2022.883308)
Supplement: Supplementary file 1 [file Table_1.DOCX]

**Sfigure legends**

**Sfigure 1.** Univariate cox regression analysis results of a single hematological marker in the training set (A) and validation set (B)

**Sfigure 2.** Results of univariate (A) and multivariate (B) cox regression analysis of AGR in the validation set
