# Supplementary material for: A Nutritional Metabolism Related Prognostic Scoring System for Patients With Newly Diagnosed Osteosarcoma
Source: Front Nutr. 2022 Apr 28;9:883308. doi: 10.3389/fnut.2022.883308 (PMC9096723; doi:10.3389/fnut.2022.883308)
Supplement: Supplementary file 2 [file Table_2.DOCX]

Supplementary table 1: Calculation formula of CONUT.

| Marker | Undernutrition degree and score | | | |
| --- | --- | --- | --- | --- |
| A.Serum albumin (g/dL) | ≥ 3.50  0 | 3.00-3.49  2 | 2.50-2.99  4 | < 2.50  6 |
| B.Total lymphocyte (count/mm^3^) | ≥ 1600  0 | 1200-1599  1 | 800-1199  2 | < 800  3 |
| C.Total cholesterol (mg/dL) | ≥ 180  0 | 140-179  1 | 100-139  2 | < 100  3 |
| CONUT | A + B + C | | | |
