# Supplementary material for: A Nutritional Metabolism Related Prognostic Scoring System for Patients With Newly Diagnosed Osteosarcoma
Source: Front Nutr. 2022 Apr 28;9:883308. doi: 10.3389/fnut.2022.883308 (PMC9096723; doi:10.3389/fnut.2022.883308)
Supplement: Supplementary file 3 [file Table_3.DOCX]

Supplementary table 2: Optimal cut-off values for 16 hematological markers

| Marker | Cutoff value |
| --- | --- |
| PNI | 50.4 |
| AGR | 1.79 |
| CONUT | 3 |
| RBC | 3.42 |
| HB | 109 |
| HCT | 0.33 |
| Total.bilirubin | 15.1 |
| Direct.bilirubin | 2.7 |
| Indirect.bilirubin | 5.1 |
| Albumin | 41.1 |
| Globulin | 23.8 |
| Glucose | 5.31 |
| Triglycerides | 0.99 |
| Cholesterol | 3.4 |
| HDL | 1.05 |
| LDL | 2.81 |
