# Supplementary figures and images for: A Nutritional Metabolism Related Prognostic Scoring System for Patients With Newly Diagnosed Osteosarcoma
Source: Front Nutr. 2022 Apr 28;9:883308. doi: 10.3389/fnut.2022.883308 (PMC9096723; doi:10.3389/fnut.2022.883308)

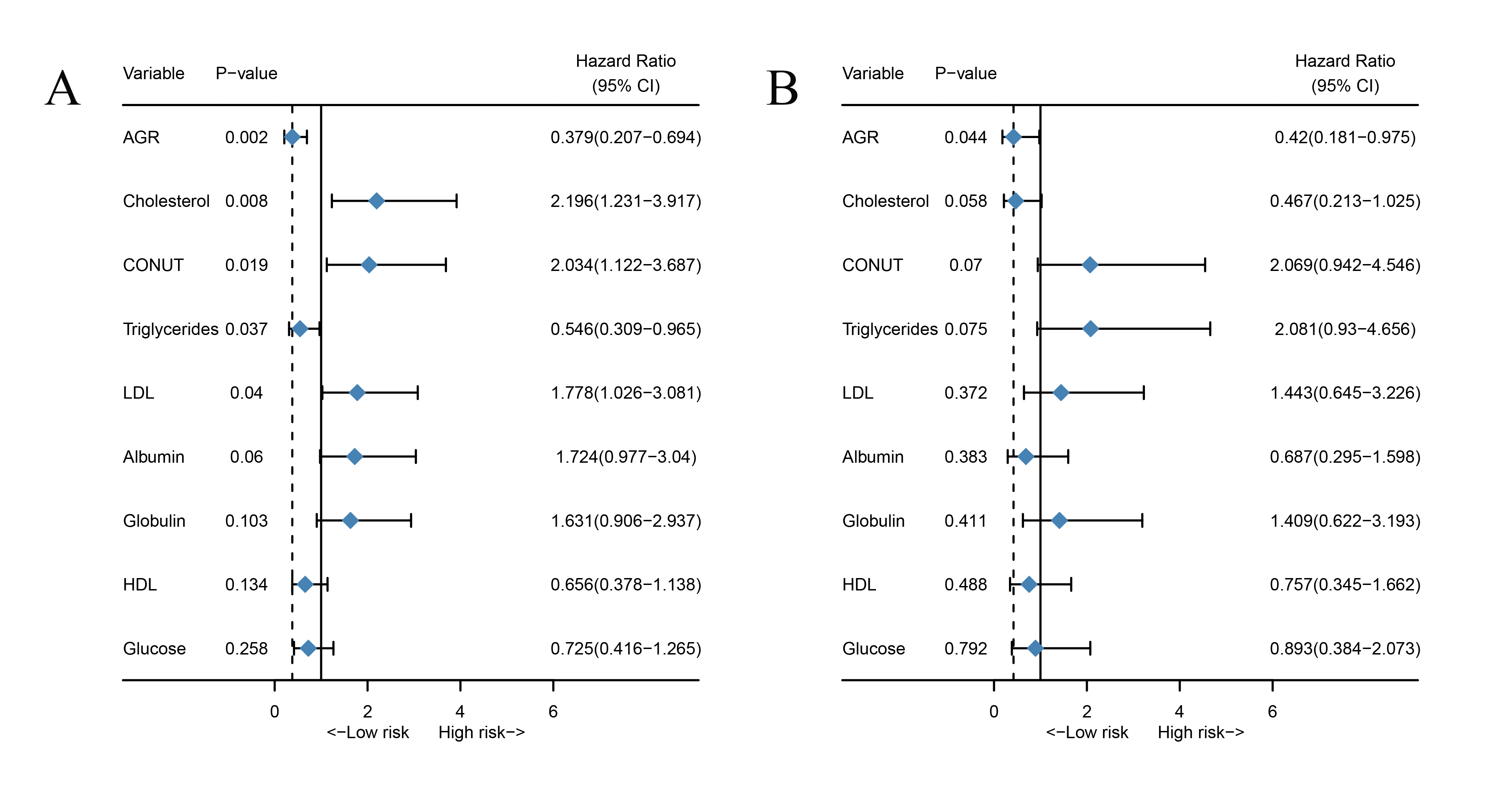

Supplement: Supplementary file 4 [file Image_1.TIF]

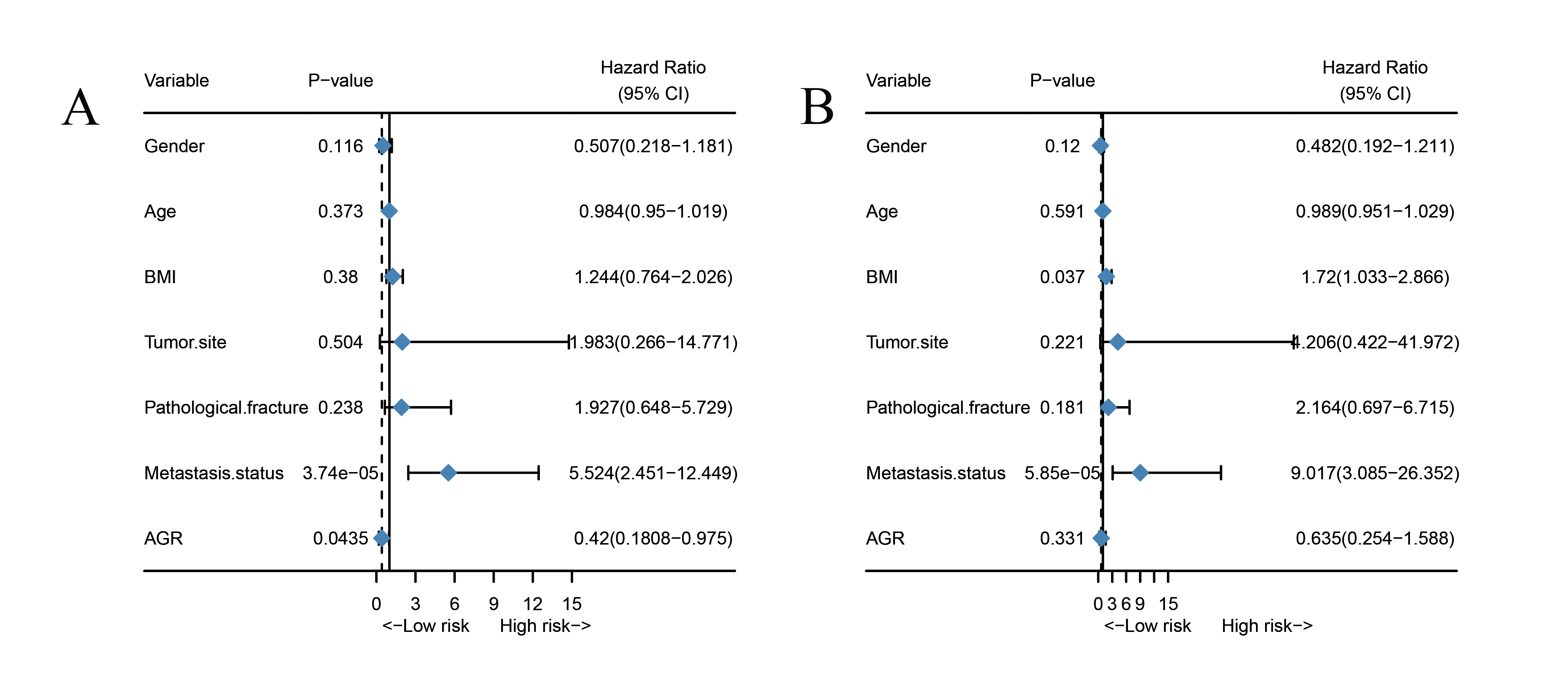

Supplement: Supplementary file 5 [file Image_2.TIF]
